# Synergistic response to climate stressors in coral is associated with genotypic variation in baseline expression

**DOI:** 10.1098/rspb.2023.2447

**Published:** 2024-03-27

**Authors:** Jenna Dilworth, Wyatt C. Million, Maria Ruggeri, Emily R. Hall, Ashley M. Dungan, Erinn M. Muller, Carly D. Kenkel

**Affiliations:** ^1^ University of Southern California, Los Angeles, CA, USA; ^2^ Mote Marine Laboratory, Sarasota, FL, USA; ^3^ The University of Melbourne, Melbourne, Victoria, Australia

**Keywords:** coral, synergistic stressors, climate change, gene expression, phenotypic plasticity, bleaching

## Abstract

As environments are rapidly reshaped due to climate change, phenotypic plasticity plays an important role in the ability of organisms to persist and is considered an especially important acclimatization mechanism for long-lived sessile organisms such as reef-building corals. Often, this ability of a single genotype to display multiple phenotypes depending on the environment is modulated by changes in gene expression, which can vary in response to environmental changes via two mechanisms: baseline expression and expression plasticity. We used transcriptome-wide expression profiling of eleven genotypes of common-gardened *Acropora cervicornis* to explore genotypic variation in the expression response to thermal and acidification stress, both individually and in combination. We show that the combination of these two stressors elicits a synergistic gene expression response, and that both baseline expression and expression plasticity in response to stress show genotypic variation. Additionally, we demonstrate that frontloading of a large module of coexpressed genes is associated with greater retention of algal symbionts under combined stress. These results illustrate that variation in the gene expression response of individuals to climate change stressors can persist even when individuals have shared environmental histories, affecting their performance under future climate change scenarios.

## 1. Introduction

Climate change is rapidly shifting environmental conditions, often beyond the physiological limits of organisms. As sessile organisms living near their upper thermal limits, reef-building corals are particularly susceptible to these shifts. Since tropical coral reefs are hotspots for marine diversity [1], these foundational reef-builders can serve as sentinel species to study the effects of climate change from the organism to ecosystem level. Scleractinian corals form obligate endosymbiotic relationships with algae in the family Symbiodiniaceae [2]. This relationship can be disrupted at just 1–2°C above mean summer sea surface temperatures in a process known as bleaching [3], often leading to mortality [4,5] or impacts on growth and fecundity [6,7]. Due to the increasing frequency of marine heatwaves, mass coral bleaching events are projected to become the norm annually by mid-century [8,9]. As such, many studies have focused on the response of reef-building corals to thermal stress alone [10–12]. However, as corals are calcifying organisms that build aragonite skeletons, lower seawater pH and aragonite saturation due to ocean and coastal acidification [9] represent an additional drain on the energy required to maintain homeostasis [13,14]. Thus, understanding how corals respond to the interactive effects of temperature and acidification will be essential to more accurately predict their future persistence in a changing climate [10].

In this context of a rapidly changing environment, phenotypic plasticity plays a key role in the ability of organisms to persist under more physiologically stressful conditions [15,16]. Often, the ability of a genotype to exhibit different phenotypes in different environmental conditions is mediated through changes in gene expression [17], which represent an important link between genetic processes and cellular and physiological responses to environmental stressors [18]. Rivera and colleagues [18] propose that gene expression changes in response to environmental stress can be conceptualized as a reaction norm framework, in which expression levels in ambient conditions are compared to those in stressful conditions. Within this framework, gene expression can be modulated by modifying two variables: the baseline expression, defined as the expression under ambient conditions, and the expression plasticity, or the magnitude of change between ambient and stressful conditions. Depending on the environmental conditions, different levels of baseline expression and plasticity, and different combinations of the two, can be adaptive. For example, higher baseline expression of genes involved in the environmental stress response may involve a tradeoff between the costs of higher constitutive expression and the benefits of being able to mount a rapid response, but this tradeoff may be beneficial in highly variable environments frequently reaching threshold conditions [19]. Additionally, a larger capacity for plasticity may replace elevated baseline expression when variability is less frequent [18,20].

In many organisms, a greater degree of expression plasticity is associated with adaptive trait variation. In rainbowfish, thermally tolerant subtropical ecotypes have a higher number of plastic genes that respond to thermal stress than less resilient temperate ecotypes [21], and elevated global gene expression plasticity correlates with higher fecundity under drought conditions in rice [22]. Similarly, gene expression plasticity facilitated adaptation to ocean warming and acidification conditions in the experimental evolution of a marine copepod [23]. In corals, higher whole-genome expression plasticity was observed in stress-resilient populations when transplanted to a novel reef environment, and greater expression plasticity of environmental stress response genes was associated with lower susceptibility to a natural summer bleaching event [24].

However, reduced expression plasticity does not necessarily translate into a less adaptive response to stress, as differences in baseline expression can also play an important role. For example, previous exposure to stress can result in increased baseline expression of genes involved in the environmental stress response in yeast, resulting in decreased plasticity but improving the response to subsequent stress [25]. Both elevated and decreased baseline expression, often referred to as frontloading and dampening respectively, can be adaptive. For example, the more thermally tolerant of two closely related desert ant species has higher baseline expression of heat shock proteins [26], while the desert-dwelling species of two congeneric land snails exhibits dampened expression of heat shock proteins, presumably to avoid the fitness consequences of their continuous upregulation [27]. In corals, both frontloading of genes involved in the thermal stress response [19] and dampening of metabolic and ribosomal processing genes [28] are adaptive in populations experiencing more thermally stressful conditions. However, differences in baseline expression have predominantly been explored in the context of specific genes, rather than at the whole transcriptome level.

In corals, differences in gene expression baselines and plasticity have largely been explored independently in the context of population-level differences within species. In non-coral systems, there is evidence of a substantial amount of within-population variation in gene expression which selection could act upon if there is genetically based variation among individuals [29–31]. However, it can be difficult if not impossible to sample genetically identical individuals in different environmental conditions in many model organisms, such as *Drosophila*, leading to a reliance on relatedness for inferring genetic effects on plasticity [31]. In addition to their utility as climate change sentinels, reef-building corals are emerging models for plasticity because clonal replicates of individual genotypes can be readily produced through fragmentation [32]. Recent work has shown that coral gene expression plasticity in response to thermal stress varies at the genotype level [33], but the link between this variation and adaptive responses to stress has not yet been established. No studies have investigated variation in baseline expression among coral genotypes, although there is some evidence that genotypic variability in the gene expression response of tropical plants to dehydration stress could be due to variation in constitutive expression [34].

Here, we use transcriptome-wide gene expression profiling to explore genotype-level differences in the response to thermal and acidification stress, both individually and combined, in the threatened Caribbean coral *Acropora cervicornis*. This study examines the response of eleven genetically distinct individuals which were common-gardened for three years before exposure to stress, minimizing the influence of environmental history. Additionally, in the Florida Keys, *A. cervicornis* predominantly hosts *Symbidinium fitti* as its algal symbiont [35,36], minimizing the contribution of symbiont genus to variation in thermal tolerance and allowing us to focus on coral host genetic effects. We show that the gene expression response to combined thermal and acidification stress is synergistic, recapitulating patterns observed in the physiological response to these stressors [37]. Additionally, we find that there is genotypic variation in both the whole transcriptome and stressor-specific gene expression patterns, and that the importance of baseline expression and expression plasticity for performance under stress varies depending on the stressor. These results indicate that variation in these molecular responses underlies intrapopulation variation in stress tolerance in this endangered reef-building coral.

## 2. Methods

### (a) Experimental design

A detailed description of field sampling and experimental design can be found in Muller *et al*. [37]. Briefly, twenty 5 cm fragments were collected from each of 12 putative *A. cervicornis* genotypes at Mote Marine Laboratory's offshore *in situ* nursery in the lower Florida Keys (24.56257° N, 81.40009° W) in July 2016. Fragments were brought to Mote's Elizabeth Moore International Center for Coral Reef Research and Restoration in Summerland Key for an *ex situ* experiment to assess the physiological and gene expression response of these genotypes to elevated temperature (referred to as ‘heat’) and *p*CO_2_ (referred to as ‘pH’), both individually and in a combined stress treatment.

After collection, fragments were acclimated to ambient conditions in Mote's Climate and Acidification Ocean Simulator (CAOS) system for one week before ramping to experimental conditions began (over the span of two to seven days for temperature, and four days for *p*CO_2_). Five replicates of each coral genotype were exposed to one of four treatments: control (704 ± 62 µatm *p*CO_2_, 27.1 ± 0.05°C), heat (798 ± 62 µatm *p*CO_2_, 31.0 ± 0.04°C), pH (1225 ± 98 µatm *p*CO_2_, 27.0 ± 0.02°C), or combined (1412 ± 90 µatm *p*CO_2_, 31.1 ± 0.05°C), which were distributed across two raceways each containing 10 replicate tanks. Corals were exposed to experimental conditions for two months before sampling for subsequent physiological measures and RNA extraction.

### (b) Physiological phenotyping

Detailed methodologies for physiological response measures can be found in the supplementary material for Muller *et al*. [37]. Briefly, 12 phenotypic traits were measured across the algal symbiont, coral host, and holobiont throughout the experiment to assess differences in physiological response between treatments and genotypes. The buoyant weight of coral fragments was measured at the beginning and end of the experiment to calculate the corrected weight change in g/cm^2^/day. Photochemical efficiency of the algal symbionts was quantified before and after exposure to the experimental treatments to determine the change in maximum quantum yield (*F*_v_/*F*_m_) and the maximum electron transfer rate (ETR_m_). Photosynthesis to respiration rate ratios and light and dark calcification rates were measured immediately after the conclusion of the two-month experiment, after which fragments were snap frozen and sent to the University of Southern California for further processing. Here, corals were analysed for total soluble protein, prophenoloxidase (PPO), phenoloxidase (PO) and peroxidase (POX) as well as total chlorophyll concentrations and algal symbiont densities.

### (c) RNA subsampling and library preparation

Snap frozen fragments received from Mote Marine Laboratory were kept at −80°C at the University of Southern California. Prior to processing for physiology described above, approximately 2 cm^2^ of tissue was removed from the apical tip of each fragment using wire cutting pliers and stored in 1.5 ml Eppendorf tubes at −80°C until RNA extraction. Tissue subsamples were then split in half using a clean razor blade to provide approximately 1 cm^2^ of tissue for RNA extraction.

RNA was extracted using the Aurum Total RNA Mini Kit (Bio-Rad Laboratories, Inc.) with minor modifications: the final elution step used 25 µl of elution solution to optimize RNA concentrations prior to cDNA synthesis. RNA yield was measured on a Take3 plate and quality was confirmed via gel electrophoresis. 1 mg of total RNA per sample was used for tag-based RNA-seq, or TagSeq [38], with modifications for sequencing on the Illumina platform. Briefly, cDNA was synthesized from the RNA template with poly-A selection that incorporated unique oligonucleotides used to remove PCR duplicates during bioinformatic analyses. 30 ng of amplified cDNA libraries were dual-end barcoded and gel size selected following the ‘freeze-and-squeeze’ method, eluting in purified water instead of sodium acetate/EDTA buffer [39]. Final libraries were quantified with qPCR and pooled in equal amounts prior to sequencing.

### (d) Bioinformatic processing

In December 2020, a total of 191 libraries were sequenced on the Illumina NextSeq High Output SR 75 over three runs at the University of Southern California Genome Core Facility. Although a total of 240 fragments were collected in July 2016, two were lost during the experiment due to mortality, 27 were lost due to thawing during an accidental freezer defrost, and 20 were discarded during sample preparation due to poor yield. Overall, 1.255 billion raw reads were generated, with individual counts ranging from 140 764 to 30.18 million per sample (median = 5.56 million reads). PCR duplicates were discarded and leader sequences trimmed from the remaining reads using custom perl scripts as described by Kenkel & Matz [24]. The fastx_toolkit (http://hannonlab.cshl.edu/fastx_toolkit) was used to remove poly-A tails (≥8 bases) targeted during cDNA synthesis steps to retain reads with a minimum sequence length of 20 bases and for quality filtering, only maintaining reads with 70% of bases with PHRED scores of at least 33. A total of 80 690 to 14.97 million reads per sample (median = 2.56 million reads) remained after quality filtering. Filtered reads were competitively mapped to a concatenated *A. cervicornis* (coral host) [40] and *Symbiodinium* (algal symbiont) [41] reference transcriptome with gmapper in SHRiMP [39]. The raw *A. cervicornis* holobiont assembly was filtered to produce a host-specific reference using a series of BLAST searches against the *Acropora digitifera* [42] and *Symbiodinium kawagutii* [43] proteomes, and the UniProt Knowledgebase Swiss-Prot database [44] following Kenkel & Bay [45]. Annotation was performed following the protocols and scripts described at https://github.com/ckenkel/annotatingTranscriptomes. Contigs were assigned putative gene names and gene ontologies using a BLASTx search (E value ≤ 10^–4^) against the UniProt Knowledgebase Swiss-Prot database [46]. A custom perl script [24] was used to group counts of sequences putatively originating from the same gene or with sufficiently high sequence similarity to justify the assumption that they serve the same function into isogroups. Reads mapping to multiple isogroups were discarded. This count file was split into host-specific and Symbiodiniaceae-specific isogroup files for subsequent analyses. One sample had no reads mapped and was removed from the counts file. In total, 55 313–10.8 million unique reads per sample (median = 2.3 million reads) mapped to 34 842 host isogroups with an average mapping efficiency of 76%. The dominance of *Symbiodinium* spp. in all experimental fragments was confirmed by mapping tag-based RNAseq libraries generated to a combination of the *A. cervicornis* reference transcriptome [40] and representative symbiont genomes for the four dominant Symbiodiniaceae genera in this region*: Symbiodinium microadriaticum* (formerly clade A) [38], *Breviolum minutum* (formerly clade B) [47], *Cladocopium* spp. (formerly clade C1) [48] and *Durusdinium trenchii* (formerly clade D) [49]. The relative proportion of reads with highly unique matches (mapping quality 40 or better) to each genome was used as a proxy for the relative abundance of symbiont genera hosted by sample (electronic supplementary material, figure S3) following Manzello *et al*. [50].

### (e) Statistical analyses

All statistical analyses were performed in R (v 4.2.1) [51]. Low-expression isogroups with less than 10 counts across more than 90% of samples were removed, retaining 16,329 highly expressed isogroups. Counts were regularized and log-transformed in DESeq2 [52] and filtered for outliers (electronic supplementary material, file S1). The remaining sample sizes were *n* = 53 for control, *n* = 47 for the heat treatment, *n* = 39 for the pH treatment and *n* = 49 for the combined stress treatment. SNP genotyping using STAGdb revealed that two of the genets, G62 and G63, were actually clones [53], so all G63 samples were re-coded as G62. Weighted gene co-expression network analysis (WGCNA) [54] was then used to identify modules of genes whose expression correlated with experimental treatments and measured physiological traits. A signed network was constructed using a soft threshold power of 14, a minimum module size of 35, and no module merging. Module-treatment and module-trait relationships were calculated using a Pearson correlation. In order to explore synergistic effects of stress, median module eigengene expression in each of the treatments was calculated and compared to control expression for six of the top 15 modules that showed significant correlations with all four treatments. To test for significant synergistic effects, the expected additive median eigengene expression of each module in the pH and heat treatments compared to the control was calculated and used as the mu (expected median) in a one-sample Wilcoxon signed-rank test. A one-tailed test was used to determine whether the observed change in median eigengene expression in the combined treatment was of significantly greater magnitude than the calculated expected median. We focus on the largest modules as many genes in corals are still unannotated [55], meaning that larger modules are more likely to contain a higher number of annotated genes that allowed us to infer potential functions of the genes in the module via subsequent analyses. The three modules with the strongest treatment correlations (yellow, purple and midnightblue) were explored in more depth. We focused on the modules that were strongly correlated with all treatments but showed the strongest correlation with one treatment in particular to narrow down our analyses on treatment-specific effects. The midnightblue module was significantly correlated with all treatments but showed the strongest correlation with the heat treatment. Both the yellow and purple modules were significantly correlated with all treatments but showed the strongest relationship with the combined treatment. GO_MWU [56] was used to perform functional enrichment analysis on these same modules using a Mann–Whitney *U*-test on the gene module membership score (kME).

DESeq2 [52] was used to extract genes differentially expressed by the various genotypes in response to combined and heat treatments, both in terms of the whole transcriptome and module-specific (yellow and purple for combined, midnightblue for heat) expression response. Differentially expressed genes were identified using a model that defined fixed factors of treatment (control versus heat, or control versus combined) and genotype. For each focal treatment, differential expression by the interaction between treatment and genotype was also identified via a model that defined a new fixed factor combining treatment and genotype (i.e. G01Heat – G62Heat, G01Control – G62Control, and G01Combined-G62Combined) following Strader and Quigley [57]. Variance stabilized counts data were output and the significance of each effect was determined using the adonis function [58]. Additionally, discriminant analysis of principal components (DAPC) was performed using the package adegenet [59] to further analyse the variance associated with each fixed effect. The distribution of samples along the DAPC axis that distinguished the response to each treatment was used to extract genotype-specific expression patterns: the median of the control distribution of each genotype was calculated as a measure of baseline expression, while the distance between individual samples in the treatment distribution and the median of the control distribution was calculated as a measure of expression plasticity. This resulted in an average baseline expression value across samples for each genotype and an individual, sample-specific plasticity value within genotypes. A linear regression was performed to determine the relationship between genotype-specific baseline expression and plasticity and the Symbiodiniaceae density retained after treatment, calculated as the difference in Symbiodiniaceae density retained by each treatment sample and the mean of the control samples of the same genotype (electronic supplementary material, figure S6).

## 3. Results

### (a) Synergistic gene expression response to combined climate change stressors

**Figure 1.**
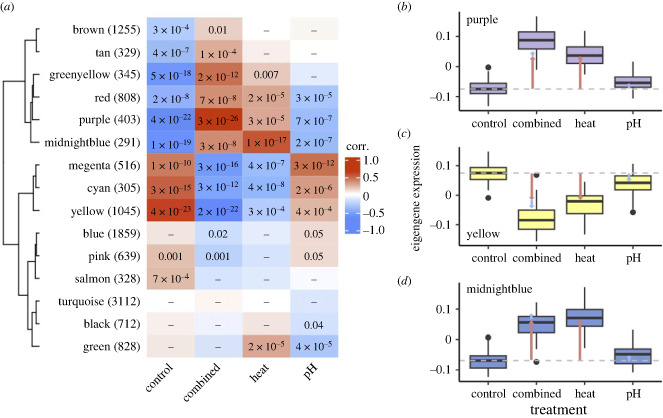
Synergistic gene expression response to combined stress. (*a*) Clustered heatmap of the 15 largest gene modules identified via weighted gene correlation network analysis (WGCNA) and their correlation with the experimental treatments—colour labels on the *y*-axis are randomly generated names for these modules, numbers in parentheses are module size. Module clusters were identified via a distance matrix computed with hclust using module eigengenes. Correlation values range from −1 (blue) to +1 (red), significant *p*-values (≤0.05) are shown in bold. (*b*–*d*) Boxplots showing median and interquartile range of module eigengene expression in each treatment for the three modules most strongly correlated with treatments: purple (*b*), yellow (*c*), and midnightblue (*d*). Grey horizontal dashed lines indicate the median eigengene expression in the control treatment, coloured arrows indicate the magnitude of change in median eigengene expression from the control to heat (red arrows) and pH (blue arrows) treatments. Stacked blue and red arrows show the expected effect based solely on additive effects of the combined stressors.

We used weighted gene correlation network analysis (WGCNA) to explore the correlation between gene expression patterns and continuous physiological trait measures as well as the effects of categorical experimental treatments. All but two of the 41 total gene modules identified showed strong, significant correlations with at least one trait or treatment (electronic supplementary material, figure S1). All physiological correlations except photosynthesis:respiration ratios and proteins involved in the host immune response (POX, PPO and PO) mirrored module correlations with treatments, suggesting these relationships can be explained by the response in physiological traits to treatment (electronic supplementary material, figure S1) [37]. Eleven of the 15 largest modules (ranging in size from 291 to 3112 co-expressed genes) strongly correlated with experimental treatment and the majority exhibited a common pattern of association: modules which negatively correlated with the heat treatment (e.g. module yellow, figure 1*c*) were positively correlated with the pH treatment, and vice versa (e.g. module purple, figure 1*b*). However, the strongest correlations with treatment in either direction were usually observed with the combined treatment, which generally showed the same direction of correlation as the heat treatment (e.g. modules yellow and purple, figure 1).

To further explore expression response to treatment, we examined module eigengene expression. The eigengene is the first principal component of the expression matrix, which is representative of the overall expression profile for genes within a module. Six of the 15 largest modules showed significant correlations with all treatments (figure 1*a*). Of these, module cyan showed only a slightly stronger response in the combined as compared to the heat and pH treatments (electronic supplementary material, figure S2*a*, *p* = 1), and three modules showed a synergistic response to the combined treatment (modules purple [*p* = 8.88 × 10^−15^] and yellow [*p* = 9.52 × 10^−13^], figures 1*b,c*; and module magenta [*p* = 1.02 × 10^−11^], electronic supplementary material, figure S2*c*) in which the magnitude of change between the control (baseline) expression and the combined treatment expression was greater than the additive changes in response to the heat and pH treatments individually. This was the case for modules both positively (i.e. upregulated; figure 1*b*) and negatively correlated (i.e. downregulated, figure 1*c*) with the combined treatment. The strongest synergies in the combined treatment were observed for the upregulated purple module, enriched for genes involved in metabolic processes and cell adhesion (figure 1*a*; electronic supplementary material, table S1), and the downregulated yellow module, enriched for genes involved in ion and transmembrane transport and cell migration (figure 1*b*; electronic supplementary material, table S1). These expression patterns recapitulate the physiological response of these corals to the experimental treatments, whereby the combined treatment had a synergistic negative effect on physiological performance as compared to the heat or pH treatments alone [37]. Two exceptions to this general pattern of synergistic responses in the combined treatment were the midnightblue module (figure 1*d*, *p* = 1), enriched for genes involved in the innate immune response (electronic supplementary material, table S1), and the red module (*p* = 1), enriched for genes involved in cell division (electronic supplementary material, table S2), which both showed the strongest upregulation under heat treatment (figure 1*a,d*; electronic supplementary material, figure S2*b*).

### (b) Genotypic variation in gene expression differs in response to heat versus combined stress

Given the strong module-specific responses to heat and combined stress revealed via WGCNA, we used DESeq2 to determine the relative importance of genotypic versus treatment effects in driving patterns of variation in response to these two treatments. In addition to examining the transcriptome-wide response, we also investigated module-specific responses of the most strongly correlated modules in each treatment: module midnightblue for heat and modules yellow and purple for combined. Genotype differences accounted for 19.7% of the variance in transcriptome-wide expression response to heat stress as compared to 7.6% accounted for by treatment (*p*_adonis_ < 0.001). Similarly, in response to combined stress there was a much stronger effect of genotype on transcriptome-wide gene expression (21.3%) than treatment (12.4%) (*p*_adonis_ < 0.001).

However, while midnightblue module-specific expression in the heat treatment showed similar patterns as the transcriptome-wide response, with genotype accounting for a greater amount of variation than treatment (22% versus 11.1%) (*p*_adonis_ < 0.001), module-specific expression in response to the combined treatment in both the yellow and purple modules varied less by genotype (purple: 15.2%, yellow: 15.6%), than by treatment (purple: 21.3%, yellow: 20%) (*p*_adonis_ < 0.001). These differences in genotypic variation of the module-specific responses between the two treatments underscore that thermal stress alone and the combination of heat and pH stress elicit unique gene expression responses. The thermal stress response in corals is rather well defined both within and across species, generally involving heat shock proteins and the innate immune system [60–62]. The differences between the response to heat and combined treatments observed here indicate that the combination of thermal and acidification stress may affect a broader suite of physiological parameters, and thus elicit more variation on a whole-transcriptome level.

### (c) Heat treatment: whole-transcriptome plasticity correlates with negative physiological response

**Figure 2.**
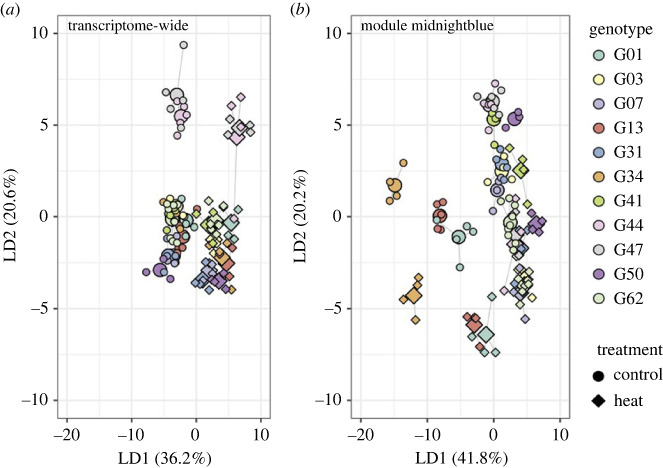
Genetically variable gene expression response to heat stress. Discriminant analysis of principal components (DAPC) of differential gene expression in response to the heat treatment. Colours represent unique genotypes and shapes represent treatment (control = 53 samples, heat = 47 samples). The enlarged centre point of each genotype/treatment cluster represents the mean. (*a*) Transcriptome-wide expression response to the heat treatment. The first linear discriminant axis separates the treatments. (*b*) Expression response to heat stress of genes in module midnightblue identified via WGCNA. The second linear discriminant axis separates the treatments.

Given the significant genotypic variation in expression both in the combined and heat treatments, we used discriminant analysis of principal components (DAPC) to determine whether changes in baseline expression or plasticity might be driving these genotypic differences in transcriptome-wide and module-specific expression. Tighter clustering was observed among genotypes in the transcriptome-wide response to heat, with the exception of genotypes 44 and 47 (figure 2*a*), whereas genotype clusters were more dispersed for midnightblue module expression (figure 2*b*). For both the transcriptome-wide and module-specific responses, the linear discriminant (LD) axis that separated out the response to treatment (LD1 for the transcriptome-wide response, LD2 for module midnightblue (figure 2)) was used to quantify genotypic differences in baseline expression (defined as the position of the median of the control distribution along the axis) and expression plasticity (defined as the distance between the median of the control distribution and position of replicate samples along the treatment axis).

**Figure 3.**
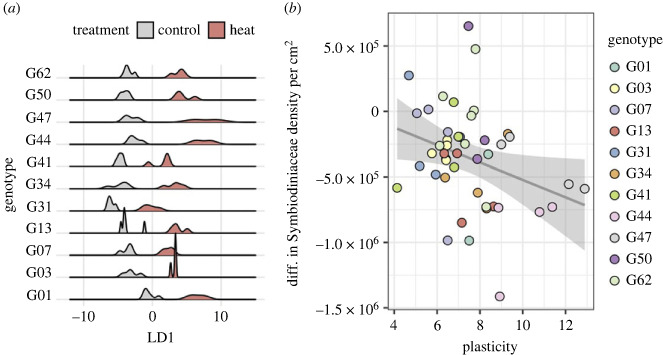
Genotypic variation in whole transcriptome expression in response to heat stress correlates with physiological performance. (*a*) Density plots showing the distribution of samples of each genotype in the control and heat treatments along LD1 from figure 2*a*. (*b*) Relationship between expression plasticity, defined as the difference between sample position along the treatment axis (LD1) and the median of the control distribution, and the difference in sample Symbiodiniaceae density per cm^2^ in heat treatment samples compared to the mean density in control treatment samples. Colours represent genotypes. Grey line represents the linear regression between plasticity and Symbiodiniaceae density (adjusted *R*^2^ = 0.07924, *p* = 0.0342). Grey shaded area represents the 95% confidence interval.

Neither baseline differences nor plasticity in the midnightblue module-specific expression response to heat treatment correlated with Symbiodiniaceae density, a metric of bleaching intensity (baseline expression: *p* = 0.421312, plasticity: see electronic supplementary material, figure S4). However, transcriptome-wide expression plasticity (figure 3*a*) was negatively correlated with the Symbiodiniaceae density retained by corals after the heat treatment (*p* = 0.0342, *R*^2^ = 0.07924) (figure 3*b*), while baseline expression was unrelated (*p* = 0.08842). The gene loadings most significantly positively associated with the heat treatment end of the transcriptome-wide treatment axis (figure 2) indicate that ribosomal genes differentiate control-level expression versus the heat response. Specifically, the gene for ribosomal protein S15a exhibited a strong, positive association with the heat response (electronic supplementary material, table S3). As increases in the expression of ribosomal proteins have previously been implicated in the early bleaching response in corals [63], the strong positive association between this ribosomal protein and the heat treatment could indicate that these corals were at the beginning of the bleaching response, and that the negative association between plasticity in these genes and Symbiodiniaceae density is capturing the initial breakdown of the symbiosis.

### (d) Combined treatment: genotypic variation in frontloading of module purple correlates with physiological response to stress

**Figure 4.**
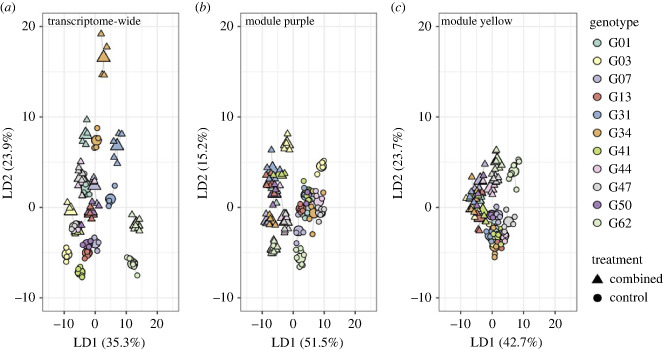
Genetically variable gene expression response to combined stress. Discriminant analysis of principal components (DAPC) of differential gene expression in response to the combined treatment. Colours represent unique genotypes and shapes represent treatment (control = 53 samples, combined = 49 samples). The enlarged center point of each genotype/treatment cluster represents the mean. (*a*) Transcriptome-wide expression response to the combined treatment. The second linear discriminant axis separates the treatments. (*b*) Expression response to the combined stress of genes in module purple identified via WGCNA. The first linear discriminant axis separates the treatments. (*c*) Expression response to the combined stress of genes in module yellow identified via WGCNA. The first linear discriminant axis separates the treatments.

In response to combined stress, the LD2 axis separated out the whole-transcriptome treatment response, which was variable by genotype (figure 4*a*). The LD1 axes differentiated treatment from control in the module purple and yellow-specific responses, which showed tighter clustering of genotypes (figure 4*b,c*). Genotypic variation in baseline expression and plasticity along the treatment axes of the transcriptome-wide (baseline: *p* = 0.0555, plasticity: see electronic supplementary material, figure S5) and yellow module-specific response (baseline: *p* = 0.314, plasticity: *p* = 0.152) to combined stress did not correlate with physiological performance.

**Figure 5.**
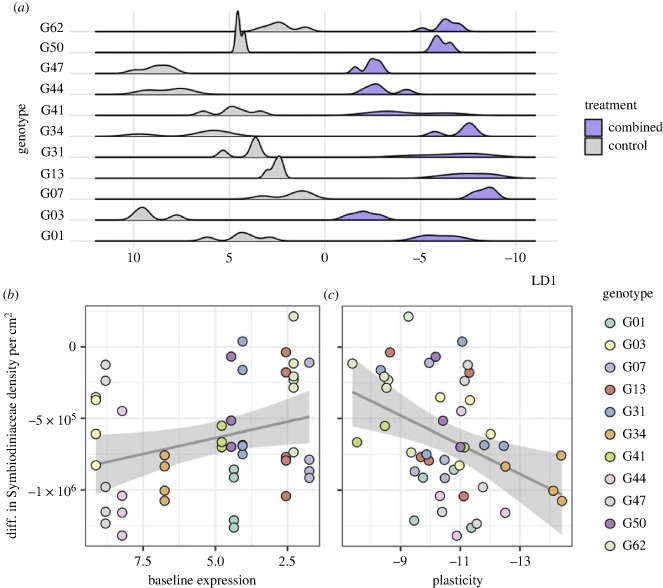
Genotypic variation in expression response of module purple to combined stress correlates with physiological performance. (*a*) Density plots showing the distribution of samples of each genotype in the control and combined treatments along LD1 from figure 4*b*. (*b*,*c*) Relationship between genotypic expression patterns and difference in Symbiodiniaceae density per cm^2^ in combined treatment samples compared to the mean density in control treatment samples. Colours represent genotypes. (*b*) Diagonal grey lines represent linear regression between baseline expression, defined as the median of the control distribution, and Symbiodiniaceae density (adjusted *R*^2^ = 0.6786, *p* = 0.04273). The *x*-axis is reversed for ease of interpretation, as lower values of baseline expression are closer to the treatment response along LD1. (*c*) Diagonal grey lines represent linear regression between expression plasticity, defined as the difference between sample position on the treatment axis and the median of the control distribution, and Symbiodiniaceae density (adjusted *R*^2^ = 0.1492, *p* = 0.00426). The *x*-axis is reversed for ease of interpretation, as plasticity is non-directional and the magnitude of plasticity is higher at the negative end of the axis. Grey shaded areas represent the 95% confidence interval.

In the purple module-specific response to the combined treatment, plasticity was negatively correlated with Symbiodiniaceae density (figure 5*c*) while baseline expression was positively correlated with this metric of bleaching (figure 5*b*). This is indicative of a frontloading-type response in this module being beneficial: there was a general trend in which genotypes with higher baseline expression showed a less plastic response, but retained more algal symbionts in response to combined acidification and thermal stress. Several of the gene loadings most strongly associated with the combined stress response end of the treatment axis (electronic supplementary material, table S4) were genes involved the protein phosphatase cascade, which plays an important role in the DNA damage response [64], as well as E3 ubiquitin ligase, which is strongly implicated in the cellular stress response in corals [65]. As such, higher baseline expression of these genes may be beneficial in mounting a successful response to the interactive effects of temperature and pH stress, resulting in better physiological functioning in the combined stress treatment. Additionally, one of the top 20 gene loadings most significantly associated with control levels of expression along the treatment axis was a retinol dehydrogenase (electronic supplementary material, table S4), which is upregulated in the growing tips of Caribbean *Acropora* species [66]. Thus, the importance of this gene in distinguishing between expression in control and combined stress conditions could indicate that the interactive effects of acidification and thermal stress influence colony calcification.

## 4. Discussion

Intraspecific variation in gene expression provides an avenue through which organisms can adjust to environmentally challenging conditions. Challenges can trigger stochastic expression responses, which can be followed by selection for the adaptive portions of this response if there is genetic variation underlying expression patterns [17]. Thus, variable gene expression can help organisms cope with variable environments in the short term, but could also result in long-term adaptation by unmasking phenotypic variability [67]. Differences in baseline expression and/or expression plasticity are two important mechanisms known to influence physiological responses across environments [18]. The relative importance of each mechanism likely depends on the environmental context as increased transcriptome-wide plasticity [24], as well as dampened [28] or frontloaded [19] baseline expression of specific genes can be adaptive at the population level. Here, we show that corals mount a synergistic gene expression response to combined ocean acidification and thermal stress, and that genotype level variation in frontloaded expression of a specific gene set (module purple) is correlated with increased retention of Symbiodiniaceae density in response to that stress. As this increased baseline expression is paired with decreased plasticity in the more successful genotypes, we find evidence for frontloading of a large suite of genes that is beneficial for individual genotype performance under climate change stress. Notably, this module was significantly enriched for cell adhesion, and genes involved in this process have been previously found to be frontloaded in more thermally tolerant coral populations [19]. Similar patterns of reduced plasticity and frontloading of genes responsive to stress have also been found in chimeric corals in which one colony is composed of more than one genotype. These chimeras demonstrated higher survival after transplantation to a more shallow environment, a stressful environmental change due to increased light and temperature [68]. The frontloaded genes in this population of chimeras were significantly enriched for metabolic processes, as was the purple module that was frontloaded by more successful genotypes in our study.

In general, frontloading of individual genes, as seen here in the purple module, is associated with lower plasticity. If this frontloading is advantageous, it can be indicative of genetic assimilation if the ancestral state was more plastic [16]. However, this evolutionary tradeoff between increased baseline expression and expression plasticity may also be maladaptive. In two allopatric congeneric oyster species, the less thermally tolerant population had higher baseline expression of heat shock proteins under ambient conditions, which was paired with lower plasticity in their thermal stress response as compared to their more thermally tolerant southern counterparts, a fixed difference that was maintained even when northern oysters were transplanted to warmer environments [69]. A loss of plasticity was also observed after several generations of selection in warming and acidification conditions in a marine copepod, which resulted in decreased performance when they were exposed to their ancestral conditions [23]. Thus, selection leading to a loss of plasticity could lead to populations less resilient to further environmental changes, as plasticity is often beneficial in variable environments. Although increased baseline expression and reduced plasticity of the purple module was advantageous in the context of interacting thermal and acidification stress, these expression dynamics could be less favoured in other environmental conditions. This specificity of the benefits of certain expression patterns is underscored by the results of this study, as this beneficial pattern of frontloading was only found in the combined stress treatment, but was not observed in response to heat stress alone.

Indeed, there is often a large stochastic component to variability in gene expression responses to stress, as stress-driven gene expression is inherently noisy [17]. Often, genes that are up- or downregulated in response to a specific stressor do not have a direct functional link to the response to that stress, meaning that a large proportion of the gene expression response is neutrally non-adaptive [17]. However, there may also be an important role of non-adaptive plasticity in evolution, as greater phenotypic variability generated by this plasticity is predicted to accelerate the strength of directional selection [70]. In a study examining gene expression patterns in guppy sibling groups transplanted into more benign, predator cue-free environments, situationally non-adaptive expression plasticity was under stronger selection than adaptive plasticity [67]. As such, our finding of the non-adaptive pattern of higher levels of whole-genome plasticity associated with reduced Symbiodiniaceae density in response to the heat treatment could still serve an important role in the evolution of an improved heat stress response, provided surviving genets can successfully reproduce.

Gene expression is dynamic and rather than fixed differences in plasticity, we may have captured genotypic differences in timing of the stress response, such that certain genotypes had already begun upregulating stress-related genes and expelling Symbiodiniaceae, while others were not yet responding to the stress in the same way. This is supported by the fact that genes previously implicated in the coral stress response, such as E3 ubiquitin ligase [65], were significantly associated with the heat response in our analyses. Additionally, there was more genotypic variation in the physiological response to stress as measured by Symbiodiniaceae density in the heat treatment than in the combined treatment, another indication that sampling may have captured genotypic variation in stress response timing. A similar pattern was observed in *Drosophila*, where cold-stress induced gene expression varied by genotype, but cold-hardy lines did not have higher baseline expression or more plasticity [71]. Rather, variation in gene expression was correlated with cold tolerance in a non-adaptive manner, indicating it may have been a symptom of cold injury rather than an adaptive response influencing cold tolerance [71]. This closely resembles the relationship between transcriptome-wide expression plasticity and Symbiodiniaceae density we observed in the heat treatment, indicating that this was more indicative of distinct physiological underpinnings of the response to thermal stress. A similar phenomenon has been observed among individual coral genotypes that are more tolerant to disease: *Acropora millepora* genotypes that were exposed to a bacterial challenge and experienced higher mortality rates mounted a stronger gene expression response than lower mortality genotypes [72]. Similarly to genotypes in our study, more robust corals remained more similar to the control condition.

Despite these patterns in plasticity observed under thermal stress alone, the combination of heat and acidification stress elicited a stronger response than just the combined effects of temperature and pH stress individually, indicating that when they co-occur, these climate change stressors elicit a synergistic, multi-layered stress response across genotypes. This synergy is particularly interesting given that module responses to individual thermal and acidification stresses showed opposing patterns. However, the coral stress response can vary depending on stress intensity. In a meta-analysis of 600 acroporid gene expression profiles, Dixon *et al.* [61] showed that gene expression responses to stress differ based on intensity, and that responses to high intensity and low intensity stressors are broadly opposite of one another. Given the weaker physiological impact of the acidification treatment in this experiment, it may represent a lower intensity stress inducing an opposing transcriptional response when compared to the higher intensity stress of the heat treatment, but the combination represents an even higher intensity challenge. This synergistic response recapitulates patterns observed by Muller *et al.* [37] in physiological traits such as buoyant weight and Symbiodiniaceae cell density in this experiment. Additionally, given the large number of gene modules we identified that showed a synergistic response to the combined treatment, it may be that the underlying molecular mechanisms governing physiological responses that did not show a synergistic response to combined thermal and acidification stress in Muller *et al*. [37], such as calcification or photosynthesis : respiration ratios, were indeed showing synergistic gene expression, but that this was not necessarily translated into synergistic effects in these higher-level traits. Indeed, the module-trait correlations between almost all physiological parameters measured in this study closely mirrored module-treatment correlations, further supporting this conclusion.

In addition to the synergism identified within the combined treatment scenario, our results demonstrate that there is variation in gene expression patterns in response to stress at the genotype level in coral, and that genotypic differences in frontloading of an entire suite of genes lead to greater retention of algal endosymbionts under combined thermal and acidification stress. Significantly, these genotypic differences were found in individuals that had been common-gardened and harboured the same algal symbiont species, minimizing environmental and symbiotic contributions to these differences. These differences in gene expression represent an important source of variation for selection to act upon, and have important implications for the evolvability of coral responses to future climate change stressors.

## Data Availability

Raw sequencing data generated for this study is available at NCBI under BioProject PRJNA992118. All input data files, including processed sequencing data, and analytical code is available at http://dx.doi.org/10.5281/zenodo.8284701 [73]. Supplementary material is available online [74].
